# Unexpected Reaction Pathway of the Alpha-Aminoalkyl Radical Derived from One-Electron Oxidation of *S*-Alkylglutathiones

**DOI:** 10.3390/molecules25040877

**Published:** 2020-02-17

**Authors:** Tomasz Pedzinski, Krzysztof Bobrowski, Bronislaw Marciniak, Piotr Filipiak

**Affiliations:** 1 Faculty of Chemistry, Adam Mickiewicz University, 61-614 Poznan, Poland; tomekp@amu.edu.pl (T.P.); marcinia@amu.edu.pl (B.M.); 2 Center for Advanced Technology, Adam Mickiewicz University, 61-614 Poznan, Poland; 3 Centre of Radiation Research and Technology, Institute of Nuclear Chemistry and Technology, 03-195 Warsaw, Poland; kris@ichtj.pl; 4 Notre Dame Radiation Laboratory, University of Notre Dame, Notre Dame, IN 46556, USA

**Keywords:** *S*-alkylglutathiones, one-electron oxidation, laser flash photolysis, decarboxylation

## Abstract

Laser flash photolysis and high-resolution mass spectrometry were used to investigate the mechanism of one-electron oxidation of two *S*-alkylglutathiones using 3-carboxybenzophenone (3CB) as a photosensitizer. This report indicates an unexpected reaction pathway of the α-aminoalkyl radical cation (αN^+^) derived from the oxidation of *S*-alkylglutathiones. Instead of a common hydrolysis reaction of αN^+^ reported earlier for methionine and other sulfur-containing aminoacids and peptides, an intramolecular ring-closure reaction was found for *S*-alkylglutathiones.

## 1. Introduction

**Figure 1:**
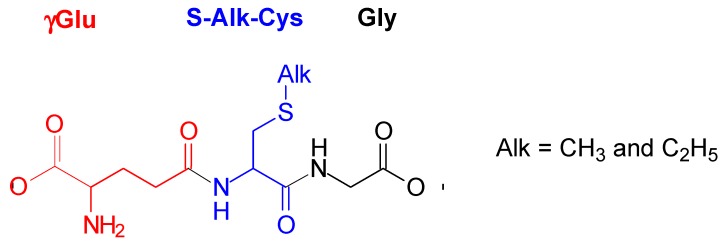
Structure of *S*-alkylglutathiones (S-Me-Glu and S-Et-Glu).

*S*-alkylglutathiones, as thioethers derived from glutathione (Glu), are considered as biologically relevant peptides (naturally occurring oligopeptides). They occur in plants, animals, and humans [1]. For instance, *S*-methylglutathione (S-Me-Glu, Figure 1) was found in yeasts [2], brain tissues [2,3], and *Escherichia coli* [4]. In biological systems, the synthesis of S-Me-Glu involves chemotaxis methyltransferase, glutathione, and *S*-adenosyl-methionine [4]. In plants and animals, S-Me-Glu was found to be a metabolic product when various methylated drugs and pesticides were present [1]. When methyl halides are metabolized by glutathione *S*-transferases, S-Me-Glu is a common product [5]. Examples are methyl chloride [6,7], methyl bromide [5,8], and methyl iodide [5,9], where erythrocytes are metabolically conjugated with Glu to form S-Me-Glu in livers, brains, and kidneys of humans and certain rodents. The biological functions of *S*-alkylglutathiones were characterized fairly extensively [1]. Some of these biological functions of *S*-alkylglutathiones are the following: (i) acting as selective ligands of brain ionotropic glutamate receptors [10], (ii) inhibiting the glyoxylase cycle [11,12], and (iii) preventing inactivation of glutathione transferase [13]. S-Me-Glu inhibits *S*-nitrosoglutathione reduction mediated by alcohol dehydrogenase, although substituting the sulfhydryl proton of Glu by a methyl moiety hardly improved the inhibitory effect of Glu alone [14]. 

Recently, antioxidant properties of *S*-ethylglutathione (S-Et-Glu) with regard to functional rat heart disorders were reported [15]. In addition, it was found that S-Me-Glu can either regulate or modulate *N*-methyl-d-aspartates glutamate receptor responses [16], can support transendothelial fluid transport [17], and can effectively promote oxidation of DNA [18]. It is also of relevance to the presence of *S*-methylglutathione in yeasts that yeasts can substitute *S*-methylcysteine for cysteine in the synthetic pathway of glutathione [3].

In the light of such wide-spread biological activities of *S*-alkylglutathiones, any oxidative degradation of them by reactive oxygen species (ROS) and carbonyl triplets is of concern during oxidative stress [17,19,20]. This is the main motivation to study free radicals produced by oxidation of *S*-alkylglutathiones and to understand the fate of these radicals. It is in these chemical studies that fundamental issues arise in how neighboring groups can affect the fate of the sulfur-centered radicals that form early in these oxidations. This is the topic of this work.

One-electron oxidation of organic sulfides, such as *S*-alkylglutathiones, typically yields a monomeric sulfur-centered radical cation (>S^●+^) as a primary product. Oxidation of the sulfur moiety is usually followed by the deprotonation at one of the two carbon atoms located in the α position to the sulfur atom, and a relatively stable carbon-centered α-thioalkyl radical (αS) is formed. In a presence of electron-rich neighboring groups, however, this monomeric sulfur radical cation (>S^●+^) can be also efficiently stabilized by the formation of inter- and intramolecular three-electron bonded species [21]. The radical cation >S^●+^ can interact with neighboring groups, which are even located over quite long distances. This was demonstrated during (i) ^●^OH radical induced oxidation of methionine residues containing peptides [22,23,24,25,26,27,28] and (ii) photoinduced one-electron oxidation of peptides containing Met residues by the 4-CB triplet state [29,30,31]. The S-Me-Glu radical cation studied in this work has been shown before to form a nine-membered cyclic structure (see Figure S4 in SI) with the participation of an amino group on the γ-Glu residue [22,31]. This intramolecular (S∴N)-bonded radical cation decays via decarboxylation of the γ-Glu residue, yielding CO_2_ and an α-aminoalkyl radical (αN, see Figure S5 in SI for more details). On the other hand, some recent reports put the existence of the nine-membered cyclic intermediate into question, claiming that the stabilization of the sulfur radical cation is achieved through a ten-membered ring with a three-electron bond between the oxidized sulfur and an oxygen in the γ-Glu’s carboxylic group [32].

This work is mainly focused on the reaction pathways of the α-aminoalkyl radical derived from two *S*-alkylglutahiones with methyl and ethyl groups, which are formed as a result of decarboxylation of these tripeptides. This highly reducing species undergoes electron-transfer reactions (in the presence of potential oxidizing agents like benzophenone, PNAP, MV^2+^), yielding a resonance-stabilized carbocation, as reported for methionine [33]. The consecutive reactions of these carbocations and their products have never been reported to date, in peptides in particular, and they are studied here by means of high-resolution mass spectrometry coupled with HPLC and steady-state and time-resolved photolysis. 

## 2. Results

### 2.1. Laser Flash Photolysis

The transients formed in the photosensitized oxidation of peptides containing thioether group are well characterized [29,31,32,34,35,36,37,38,39]. Photosensitized oxidation of *S*-alkylglutathiones leads to numerous radical transient products [31,32]. As schematically presented in Scheme SI in Supplementary Materials, the 355 nm laser photolysis of the investigated system leads to the formation of the 3-carboxybenzophenone triplet state (3CB*), which is collisionally quenched by either S-Me-Glu or S-Et-Glu, yielding 3-carboxybenzophenone radical anion (3CB^●–^) (or 3-carboxybenzophenone ketyl radical (3CBH^●^), pK_a_ = 9.5 [37]) and a monomeric S-centered radical cation (>S^●+^). After charge separation of the encounter complex, >S^●+^ is stabilized by numerous equilibria involving intermediates with (S∴O), (S∴S), and (S∴N) two-centered three-electron bonds. All these equilibria eventually lead to two irreversible reaction channels: (i) deprotonation of >S^●+^ at the carbon atoms adjacent to the sulfur yielding α-(alkylthio)alkyl radicals αS and (ii) decarboxylation via the nine-membered sulfur-nitrogen three-electron bonded transient that yields αN radicals (for more detailed mechanism see Scheme SI in Supplementary Materials).

The main intermediates involved in the formation of stable products, as detected by pulse radiolysis and/or laser flash photolysis methods, are the following: 3CB^●–^ (ketyl radical anion), αS, and αN radicals. The latter species, under laser photolysis conditions, can only be produced in basic solutions because it requires the formation of a three-electron bond between the oxidized sulfur atom, and the nitrogen atom containing a free electron pair (in neutral and acidic pH, the amino group is protonated, and consequently no free electron pair is available to form the αN precursor). These radicals can recombine forming stable products or be involved in more complex mechanisms, as described below.

**Figure 2:**
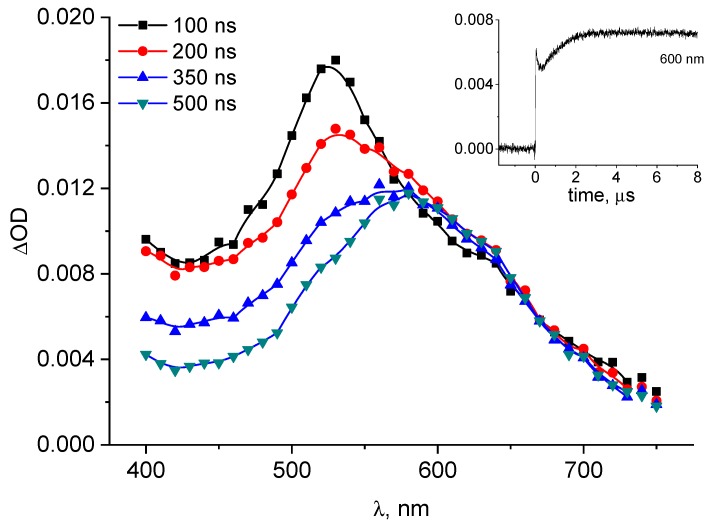
Transient absorption spectra following 3CB (4 mM) triplet photosensitized oxidation of S-Me-Glu (10 mM) at pH = 11.2. Inset: kinetics monitored at λ = 600 nm (absorption maximum of 3CB^●–^).

The transient absorption spectra following 3CB triplet quenching by S-Me-Glu (10 mM) in aqueous solution at pH = 11.2 are presented in Figure 2.

The spectra clearly show the presence of the 3CB radical anion (3CB^●–^) (with its typical absorption band characterized by λ_max_ ≈ 600 nm). Under these experimental conditions, the spectra of neither αS nor αN radicals could be recorded owing to the presence of the strongly absorbing 3CB ground state. It has been reported, however, that under pulse radiolysis conditions (in the absence of the photosensitizer) both radicals were observed with their absorptions in the UV region of 265–290 nm [40].

An indirect support for the formation of the αN radical comes from the kinetic analysis of the 3CB^●–^ formation. Its initial fast growth is the result of the electron transfer between the two reactants in the primary quenching process, leading to the formation of intermediate radical pair (encounter complex 3CB^●–^…^+●^S<), which decays mainly via charge separation, forming 3CB^●–^. The secondary, somewhat slower growth can be clearly seen in the inset to Figure 2. The nature of this slower secondary growth is attributed to an electron transfer from the αN radical (which is known to be a highly reducing species) to the ground state sensitizer (3CB) [41], which occurs on a longer timescale and can be conveniently monitored at 600 nm (inset kinetic in Figure 2). This reaction (the electron transfer between αN radical and 3CB) is shown in Scheme SI and leads to the formation of an αN carbocation (αN^+^) (stabilized by the resonance owing to the adjacent nitrogen atom) and 3CB^●–^. Moreover, the amount of 3CB^●–^ formed in the secondary reaction can be quantified based on the known molar absorption coefficient of this species (ε_600_ = 5200 M^−1^cm^−1^ [37]) and the analysis of the kinetic trace in the inset to Figure 2. The concentration of 3CB^●–^ formed in this secondary reaction was calculated to be 0.6 μM, and this value corresponds to the quantum yield of 0.24, which is in a good agreement with the quantum yield of CO_2_ (Φ_CO2_ = 0.23 reported earlier [31]). The initially formed species with maximum at 520 nm is the 3CB triplet state, decaying because of the bimolecular quenching by S-Alk-Glu (as shown in Figure S3).

It is interesting to follow the reactions of this carbocation because such a reaction mechanism has never been reported before. According to the literature, the same αN^+^ cation moiety was proposed in analogous systems when the presence of αN was probed by αN reactions with PNAP (p-nitroacetophenone) [42] and MV^2+^ [33]. In our studies, 3CB plays essentially the same role as PNAP or MV^2+^ (both are electron acceptors) in their reactions with αN. Therefore, the presence of αN^+^ in the reaction scheme (see Scheme SI for more details) is plausible. 

### 2.2. Analysis of High-Resolution Mass Spectra

**Scheme 1:**
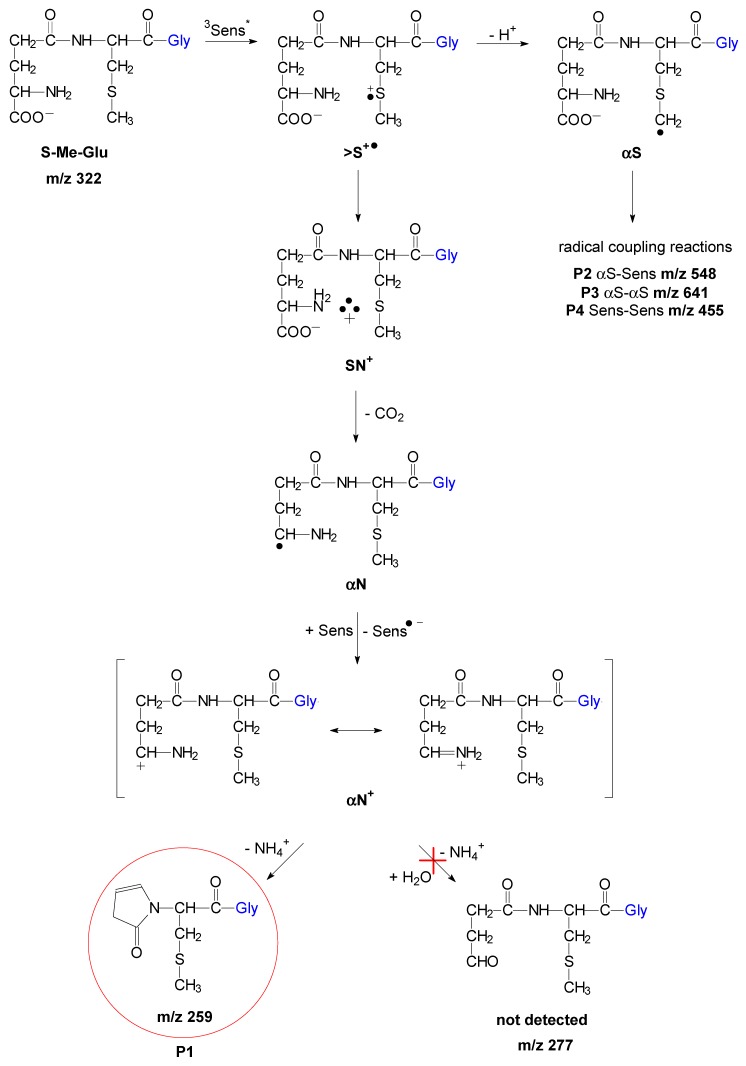
Mechanism of short-lived intermediates and stable photoproducts formation for sensitized by 3CB photooxidation of S-Me-Glu at pH 11.2 (**P2**, **P3**, and **P4** structures see Figure S6 in Supplementary Materials).

The fate of such an αN^+^ transient can be deduced from an analysis of stable products carried out by means of high-resolution mass spectrometry. In earlier studies on methionine oxidation [42], it was suggested that the oxidized amino radical derived from methionine (depicted here as αN^+^ and existing in two resonance forms as presented in Scheme 1) is subjected to hydrolysis, yielding the corresponding aldehyde and ammonia (Scheme 1). 

In the present studies, the presence of respective aldehyde was searched for by means of advanced LC-MS techniques. However, no traces of its formation were found. Interestingly, the other products were found, and the rationale for their formation is discussed in detail below.

As described above, the transients formed in the *S*-alkyl-Glu photooxidation are well characterized, but the reaction pathways leading to the stable products have not been well established. High resolution mass measurements performed on TOF (time-of-flight) analyzers allow for the determination of the molecular composition of the analyzed molecular ions (typically MH^+^) or fragment ions through the exact (monoisotopic) mass determination. One can also deduce the molecular composition of a neutral fragment loss by a simple subtraction of appropriate monoisotopic masses. 

The samples containing aqueous solutions of S-Me-Glu or S-Et-Glu (10 mM) and 3CB (2 mM) at pH = 11.2 were irradiated using a 355 nm CW laser (the excitation wavelength was chosen for selective excitation of the 3CB sensitizer molecule). The irradiated samples were subjected to further LC-MS analysis.

**Figure 3:**
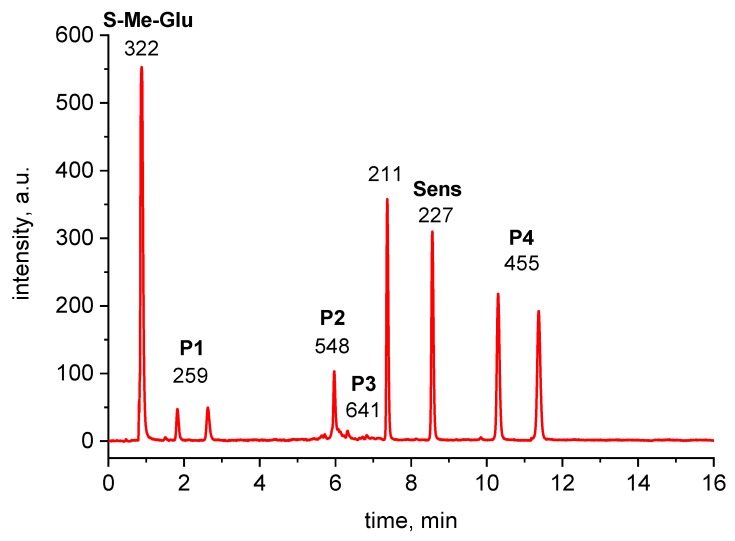
HPLC-MS chromatogram of 355 nm irradiated 3CB (2 mM) and S-Me-Glu (10 mM) mixture. Peaks are labelled with *m/z* of the most intense peak of the acquired MS spectrum. For high-resolution *m/z* values, see Table 1.

The *m/z* values were measured and interpreted for all of the chromatographic peaks presented in Figure 3.

**Figure 4:**
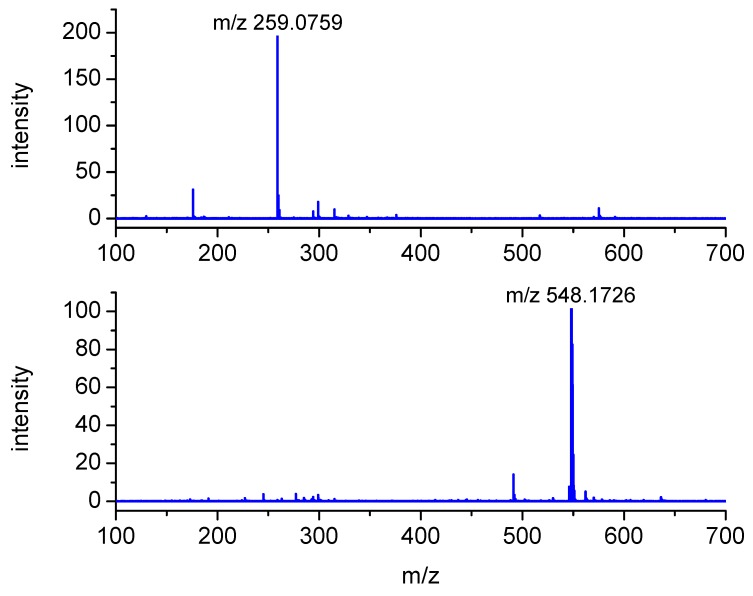
Mass spectra of the LC-MS peaks at retention times of 2.6 min (upper) and 6.0 min (lower) formed in the case of triplet 3CB-sensitized oxidation of S-Me-Glu (for more MS spectra, see Figure S2 in Supplementary Materials).

The peaks with *m/z* 322 and 227 were assigned to the substrates, S-Me-Glu and 3CB, respectively. The peaks with *m/z* 211 and 455 were assigned to the products derived from the photosensitizer 3CB and are presented in Figure 3. The peaks with *m/z* 548 and 259 are relevant to S-Me-Glu oxidation, and they will be discussed in more detail. It is noteworthy that, as expected, traces of αS-αS radical coupling products (*m/z* 641) were also detected (see small peaks at retention times of 6–7 min in Figure 3), but these photoproducts were not subjected to MSMS analysis in this work owing to their small yields. The high-resolution mass spectra of the latter two peaks are presented in Figure 4. Each MS spectrum shows one dominant peak corresponding to an MH^+^ ion, and no efficient fragmentation was observed. From *m/z* 548.1726, a molecular composition of C25H30N3O9S (MH^+^) was formulated. 

This composition can be assigned to a recombination product of two radicals: 3CB^●–^ and the carbon-centered αS radical from S-Me-Glu. Both radicals were detected earlier using time-resolved spectrophotometric detection [31] (αS in pulse radiolysis and 3CB^●–^/3CBH^●^ in laser flash photolysis). Both were shown to be relatively stable, decaying on the millisecond time domain. It is quite plausible that such C-centered radicals can diffuse together and eventually can combine to give the observed *m/z* 548 stable product (see Figure S6 Supplementary Materials).

**Figure 5:**
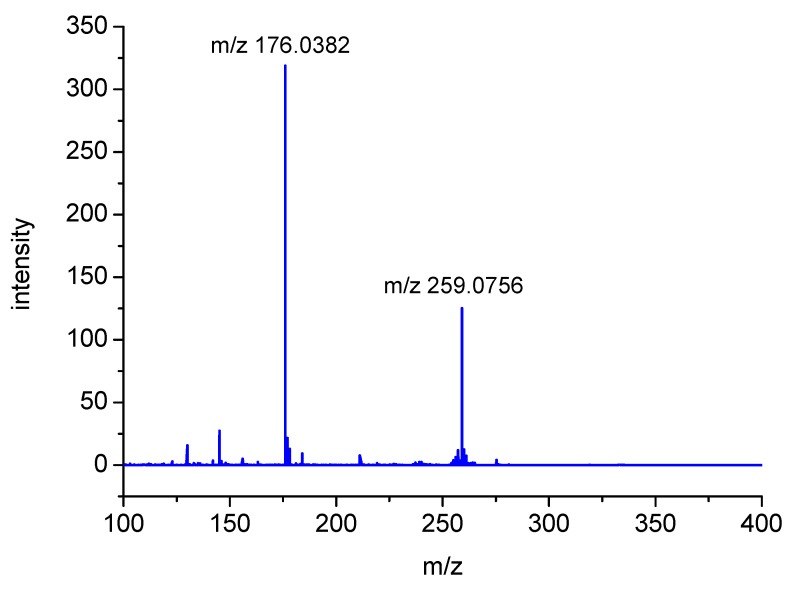
Fragmentation spectrum of *m/z* 259 ion (photoproduct **P1**) derived from oxidation of S-Me-Glu (electrospray ionization (ESI) source, capillary voltage = 4500 V, collision energy (CE) = 10 eV).

The nominal peak with *m/z* 259 (exact MH^+^ mass = 259.0759) is presented in Figure 3 chromatogram as showing up in two peaks having similar retention times (**P1**). Both peaks were assigned to a MH^+^ molecular composition of C10H15N2O4S. Moreover, the fragmentation MSMS spectrum showed an intense fragmentation peak with *m/z* 176.0382 (C6H10NO3S), as illustrated in Figure 5.

**Figure 6:**
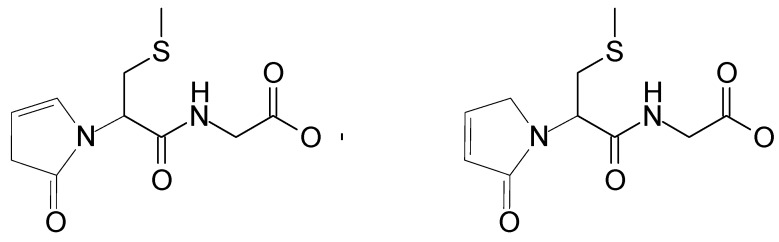
Suggested structures of isomeric products (depicted as **P1**, *m/z* 259) derived from S-Me-Glu (similar products **P’1** derived from S-Et-Glu give *m/z* 273—see Table 1).

This corresponds to the loss of a neutral species of C4H5NO. These observations suggest that these two peaks can be assigned to the isomeric structures of the same stable product (shown in Figure 6). A preliminary mechanism of its formation is presented in Scheme SI and Scheme SII (in Supplementary Materials) in details.

The exact mass of the *m/z* = 548.1726 peak corresponds to the molecular composition of C25H30N3O9S (see Table 1 for details). Such a formula suggests that this product results from the combination of the αS radical and the 3CB ketyl radical (see suggested structures in Figure S6 in Supplementary Materials).

The exact mass measurements (the calculated error is below 5 ppm) and the isotopic pattern (not shown) confirm the expected elemental composition of this product.

Similar reaction pathways were observed in the case of triplet 3CB-sensitized oxidation of S-Et-Glu, where the corresponding *m/z* values were larger by 14 owing to the additional –CH_2_– group present in this molecule (see Table 1 for MS data).

## 3. Discussion 

### Mechanism

The postulated fate of the αN^+^ carbocation is presented in Scheme SI. As already discussed above, the presence of such carbocations was suggested earlier in methionine oxidation, where they were found to react with water, eventually forming corresponding aldehyde [33]. A review paper on carbocations reactivity [43] shows that carbocations are generally good electron acceptors. However, for certain systems (even though electron transfer to the cations is favorably exothermic), the process does occur to any significant extent. As presented in Scheme SI (and more detailed in Scheme SII from the Supplementary Materials), the αN^+^ carbocation (resulting from the oxidation of the αN radical) can interact with the peptidic nitrogen, forming a five-membered ring structure. Following the formation of this cyclic structure, intramolecular proton transfer can occur between the nitrogen atoms, followed by the elimination of ammonia. In alkaline conditions (note that only at basic pH can this reaction take place), it is likely that this reaction will be followed by deprotonation, yielding the final, stable peptide modification. The product of *m/z* = 259 (or 273 for S-Et-Glu) can have two isomers because of a possible migration of the carbocation in the newly formed cyclic structure (Figure 6).

The **P1** (and **P’1**) photoproducts presented in Figure 6 were not produced efficiently after irradiation at neutral pH, and only their trace amounts were found (see chromatogram for the pH = 6 in Figure S1 in Supplementary Materials). This result is consistent with our previous observations showing that decarboxylation does not occur efficiently at pHs below the p*K*_a_ of the amino group in the γ-Glu residue of S-Me-Glu (p*K*_a_ = 8.7) [31]. In the absence of decarboxylation at pH 6, the αN radical cannot be formed, and consequently the reaction described above in Scheme SI for alkaline solutions does not occur efficiently at pH close to neutral.

Contrary to our consistent results, it was recently postulated [32] that decarboxylation proceeds via a 10-membered ring (presented in Figure S7 in Supplementary Materials) with participation of an oxygen atom in the carboxylic group and the respective (S∴O)-bonded transient as a precursor. As the carboxylic group is deprotonated at both pH values in our experiments (pH = 6.0 and 11.2), no difference in decarboxylation yields should be observed. However, our experimental results indicate the difference in the reaction pattern depending on pH in spite of the fact that the carboxyl group is deprotonated at both pHs. Following the same line of reasoning, the nitrogen atom located in the amino group is protonated at pH 6 and deprotonated at pH 11.2. This fact supports the notion that the nitrogen atom participates in the stabilization of the sulfur radical cation (a precursor for decarboxylation). 

Moreover, it has been demonstrated (e.g. with 3-methylthiopropionic acid) that the (S∴O)-bonded transients, although frequently formed in similar systems, cannot be precursors for decarboxylation. In other words, decarboxylation must be preceded by the formation of the (S∴N)^+^ transient [44].

## 4. Materials and Methods 

### 4.1. Nanosecond Laser Flash Photolysis (LFP)

Nanosecond laser flash photolysis experiments were performed using 3-carboxybenzophenone (3CB) as a photosensitizer. The setup has been described in detail elsewhere [38]. Briefly, this setup uses a Nd:YAG laser with its third harmonic 355 nm excitation wavelength as a pump (Spectra-Physics, Mountain View, CA, USA) and a pulsed Xe lamp to probe the excited sample. The laser pulse energy used in LFP experiments was set to 3 mJ (higher pulse energies could induce undesired multiphotonic processes). All flash photolysis experiments were carried out in 1 × 1 cm rectangular quartz fluorescence cells and the absorbances of solutions at 355 nm (excitation wavelength) were set to approximately A_355_ = 0.4. The concentration of the quencher (S-Alk-Glu) was 10 mM. Kinetic traces were taken between 360 and 750 at 10 nm intervals. The time-resolved absorption spectra were constructed from kinetic traces. 

### 4.2. Preparation of Solutions

All solutions were prepared in purified water. The pH of solutions was adjusted by adding potassium hydroxide and/or perchloric acid using a Mettler Toledo Five Easy FE20 pH-meter equipped with a semimicro InLab electrode from Mettler Toledo (Columbus, OH, USA). Aqueous solutions (unbuffered) were prepared shortly prior to each experiment and were deoxygenated by bubbling high-purity argon through them for at least 15 min. The solutions were stirred during the experiments. 

### 4.3. Steady-State Photolysis 

Steady-state photolysis experiments were performed in a 1 × 1 cm rectangular cell on an optical bench irradiation system using a Genesis CX355STM OPSL laser from Coherent (Santa Clara, CA, USA), with 355 nm emission wavelength (the output power used was set at 50 mW). The concentrations of 3CB and S-Alk-Glu were 4 mM and 10 mM, respectively.

### 4.4. Chemicals 

3CB, S-Me-Glu, and S-Et-Glu were purchased from Sigma-Aldrich (St. Louis, MO, USA) and used as received. Water was purified through a Millipore (Merck Milli-Q) system. High purity argon (Linde) was used to purge the freshly prepared solutions of the reagents for photolysis experiments.

### 4.5. LC-MS Measurements 

The LC-MS measurements were carried out using a liquid chromatography Thermo Scientific/Dionex Ultimate 3000 (Waltham, MA, USA) system with a quaternary pump, membrane degasser, autosampler, temperature-controlled column compartment, and diode-array detector. The LC method employs a binary gradient of acetonitrile and water with 0.1% (*v/v*) formic acid. The LC separation was carried out on a Thermo Scientific Accucore^TM^ C18 reversed-phase column (100 × 2.1 mm, 2.6 μm particle size) at a flow rate of 0.3 mL/min. This UHPLC system was coupled to a hybrid time-of-flight mass spectrometer (Impact, Bruker, Billerica, MA, USA) via an electrospray ionization (ESI) source operated in positive ion mode. Ions were generated by ESI under the following conditions: a flow rate of 0.3 mL/min, a nebulizer pressure of 1.5 bar, a spray voltage of 5200 V, and a drying gas temperature of 200 °C.

## 5. Conclusions 

In the current paper, we provide an experimental proof for reaction pathways of two major C-centered radicals formed in the photosensitized oxidation of S-Me-Glu and S-Et-Glu in the presence of 3CB. The α-thioalkyl radicals (αS) are formed as a result of deprotonation of the oxidized peptide (>S^●+^), yielding the radical coupling products **P2** and **P3**. The α-aminoalkyl radicals (αN), resulting from decarboxylation, were observed exclusively at basic pH. Its negligible amounts at neutral pH can be rationalized by the absence of its precursor (the (S∴N)^+^ radical cation). At pH = 6.0, the (S∴N)^+^ radical cation cannot be formed because of the protonation of the amino group and the lack of an electron lone pair on the nitrogen atom necessary to form a 2c-3e (S∴N) bond. In the presence of 3CB, the highly reducing α-aminoalkyl radical (αN) undergoes an electron-transfer reaction, yielding a resonance-stabilized carbocation (αN^+^). The latter species, instead of hydrolysis reaction leading to an aldehyde, undergoes an unexpected competitive intramolecular ring-closure reaction, forming stable isomeric products at *m/z* 259 (**P1**) for S-Me-Glu (and **P’1** *m/z* 273 for S-Et-Glu). This reaction pathway, leading to stable peptide modifications, is reported here for the first time.

The formation of the cyclic structure from the oxidized αN radical can be used as a probe for decarboxylation of *S*-alkylglutathiones and other peptides of a similar structure. It should be noted that, because the reaction described above can only proceed from the carbocation adjacent to the amino group (and not directly from the αN radical), there must be an oxidizing agent, such as 3CB, present in the investigated system.

As excited carbonyl compounds can be produced “in vivo” [20,45], similar reactions to those investigated in this work can potentially take place in real biological systems and for a variety of sulfur-containing peptides and proteins that exist in nature.

## Figures and Tables

**Table 1:** High-resolution MS data for S-Me-Glu (first two rows) and S-Et-Glu (last two rows).

MH^+^ Exact Mass (Measured)	MH^+^ Exact Mass (Calculated)	Most Abundant Fragment (MSMS)	Molecular Composition (MH^+^)	Product
259.0759	259.0753	176.0382	C10H15N2O4S	P1
548.1726	548.1703	-	C25H30N3O9S	P2 (αS-Sens)
273.1003	273.0909	190.0540	C11H17N2O4S	P’1
562.1864	562.1859	-	C26H32N3O9S	P’2 (αS-Sens)

For *m/z* values and MS spectra of products derived from 3CB, see Supplementary Information. **P1**/**P2** products derived from the oxidation of S-Me-Glu; **P’1**/**P’2** derived from S-Et-Glu.

## Supplementary Materials

The following are available online at https://www.mdpi.com/1420-3049/25/4/877/s1, Figure S1: HPLC-DAD chromatogram of non-irradiated (upper) and irradiated for 10 minutes (lower) 3CB (3 mM)—S-Me-Glu (10 mM) system at pH 6.0. Only traces (as compared with pH 11) of the irradiation products at *m/z* 259 can be seen at retention time of approximately 2.5 min. Figure S2: 3CB + S-Me-Glu, irradiation time 10 min—Mass spectra of the LC-MS chromatogram for all detected peaks with their retention times. Figure S3. LFP kinetic traces monitored at 520 nm (A) and 600 nm (B). The 3CB triplet state is quenched efficiently by S-Me-Glu, shortening its lifetime to approximately 100 ns ([3CB] = 4 mM, [S-Me-Glu] = 10 mM, pH = 11.2). Figure S4: Structure of a 9-membered cyclic (S∴N)-bonded radical cation. Figure S5: Structure of α-aminoalkyl radical derived from S-methylglutathione (S-Me-Glu). Scheme SI: Mechanism of primary reactions in 3CB sensitized photooxidation of S-Me-Glu at pH = 11.2. Figure S6: Suggested structures of products: P2 *m/z* 548—adduct of S-Me-Glu α-thioalkyl radical (αS) with 3CB ketyl radical; P3 *m/z* 641—αS radical dimer; P4 *m/z* 455—3CB ketyl radical dimer. Scheme SII: Suggested mechanism of cyclic product formation from the αN+ derived from S-Me-Glu. Figure S7: Structure of a 10-membered cyclic (S∴O)-bonded transient postulated in the work of [32].
