# Supplementary material for: Unexpected Reaction Pathway of the Alpha-Aminoalkyl Radical Derived from One-Electron Oxidation of S-Alkylglutathiones
Source: Molecules. 2020 Feb 17;25(4):877. doi: 10.3390/molecules25040877 (PMC7070667; doi:10.3390/molecules25040877)
Supplement: Supplementary file 1 [file molecules-25-00877-s001.pdf]

molecules

Supplementary Materials

Unexpected Reaction Pathway of the Alpha-Aminoalkyl Radical Derived from One-Electron Oxidation of S-Alkyl-Glutathiones

Tomasz Pedzinski, Krzysztof Bobrowski, Bronislaw Marciniak, and Piotr Filipiak*

*Correspondence: piotrf@amu.edu.pl; Tel: +48-61-829-1738;

Table of contents:

Figure S1	page 2
Figure S2	page 3
Figure S3	page 4
Figure S4	page 4
Figure S5	page 4
Scheme SI	page 5
Figure S6	page 6
Scheme SII	page 7
Figure S7	page 7

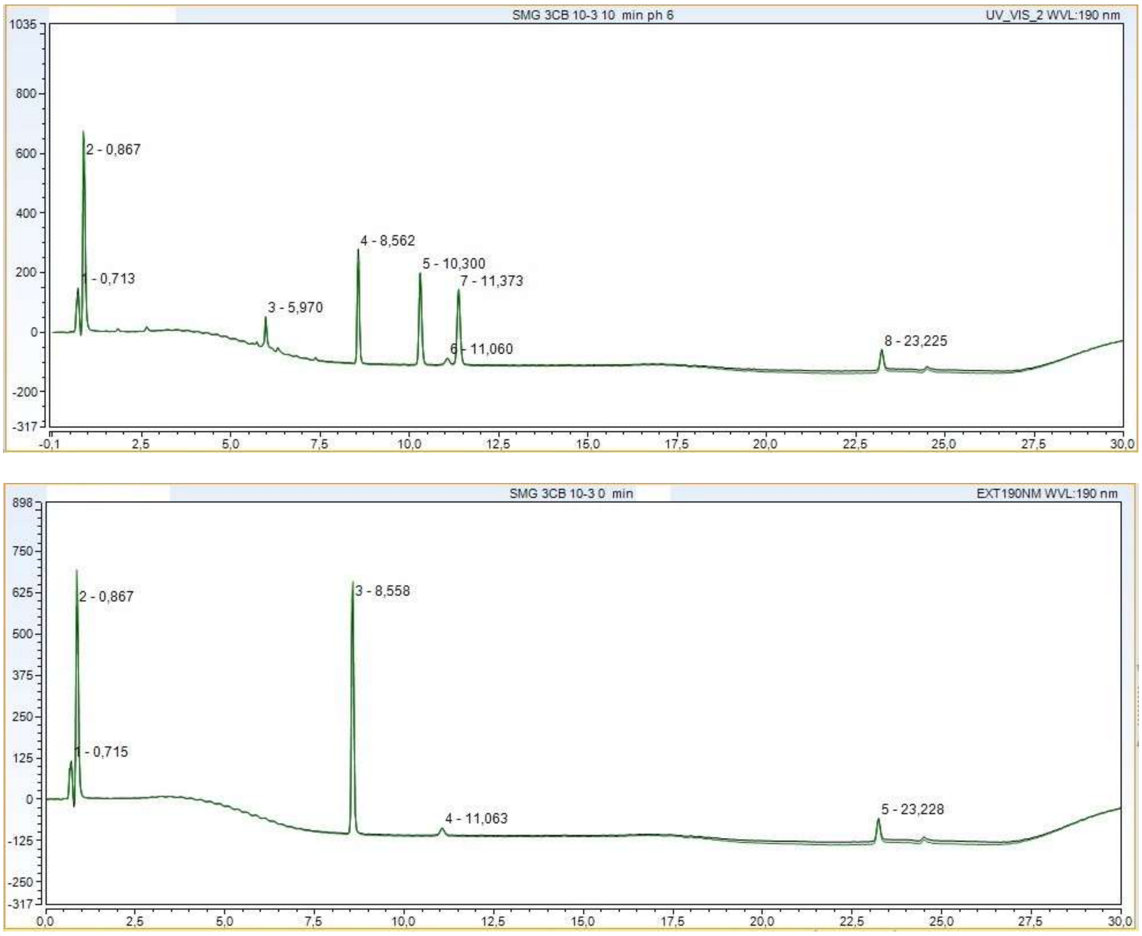

Figure S1. HPLC-DAD chromatogram of non-irradiated (upper) and irradiated for 10 minutes (lower) 3CB (3 mM) - S-Me-Glu (10 mM) system at pH 6.0. Only traces (as compared with pH 11) of the irradiation products at m/z 259 can be seen at retention time of approximately 2.5 min.

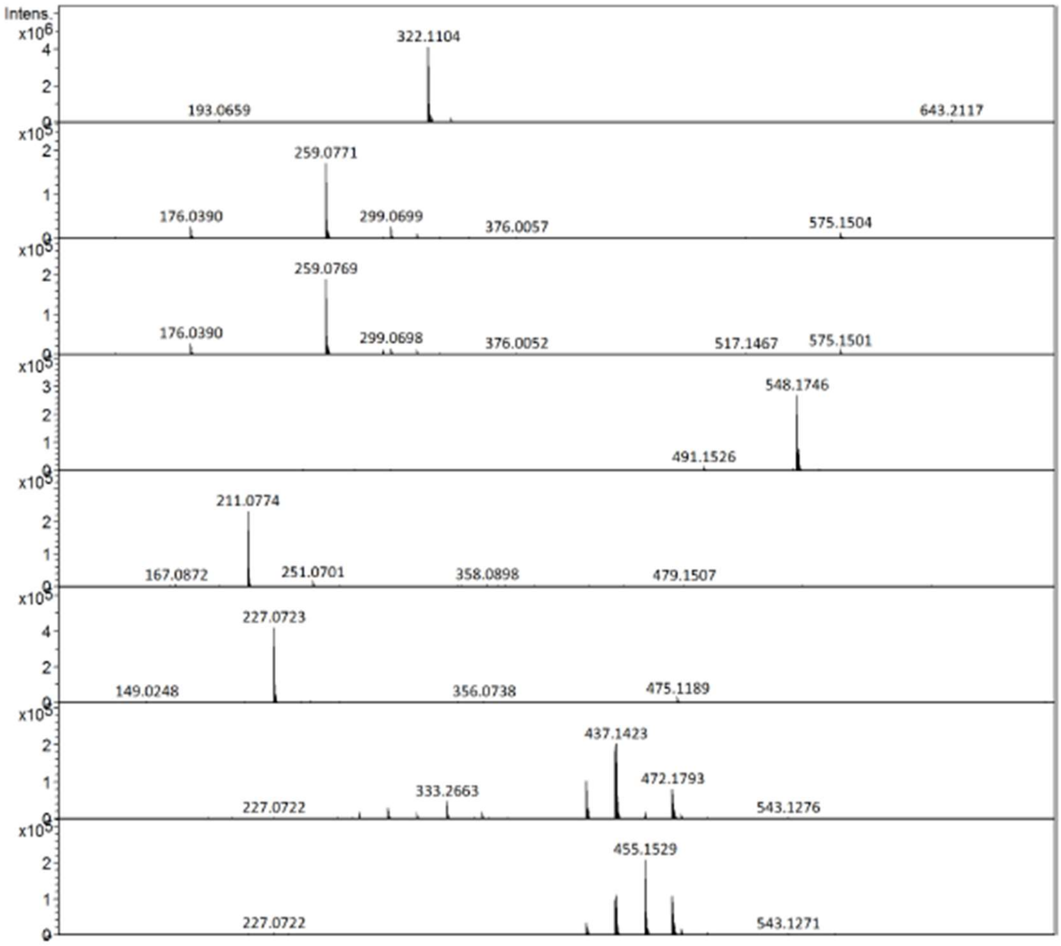

Figure S2. 3CB + S-Me-Glu, irradiation time 10 min—mass spectra of the LC-MS chromatogram for all detected peaks with their retention times.

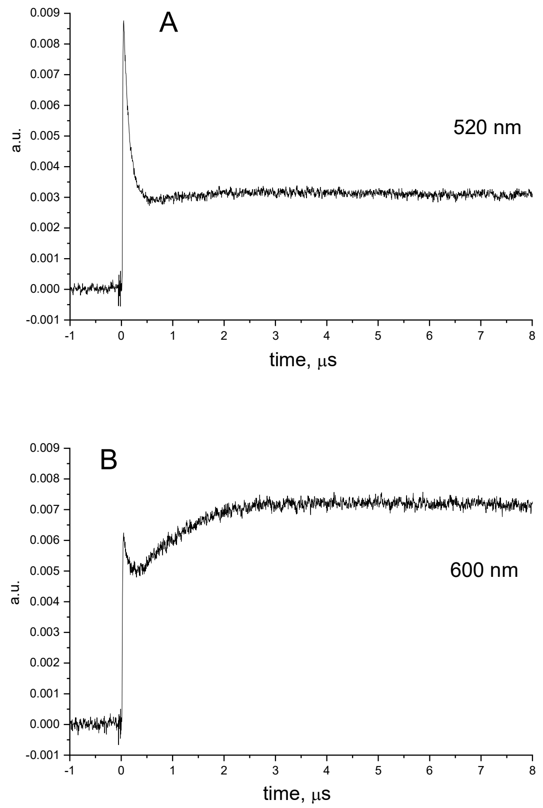

Figure S3. LFP kinetic traces monitored at 520 nm (A) and 600 nm (B). The 3CB triplet state is quenched efficiently by S-Me-Glu, shortening its lifetime to approximately 100 ns ([3CB] = 4 mM, [S-Me-Glu] = 10 mM, pH = 11.2).

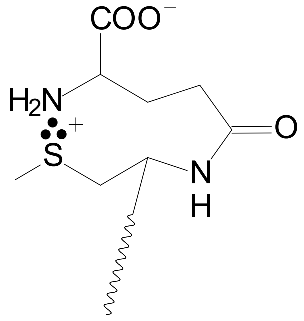

Figure S4. Structure of a 9-membered cyclic (S∴N)-bonded radical cation.

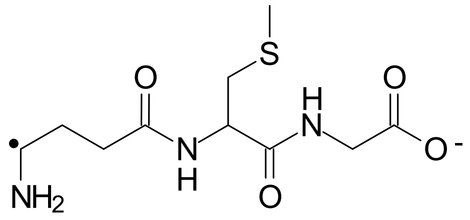

α-aminoalkyl radical (αN)

Figure S5. Structure of α-aminoalkyl radical derived from S-Methyl-Glutathione (S-Me-Glu).

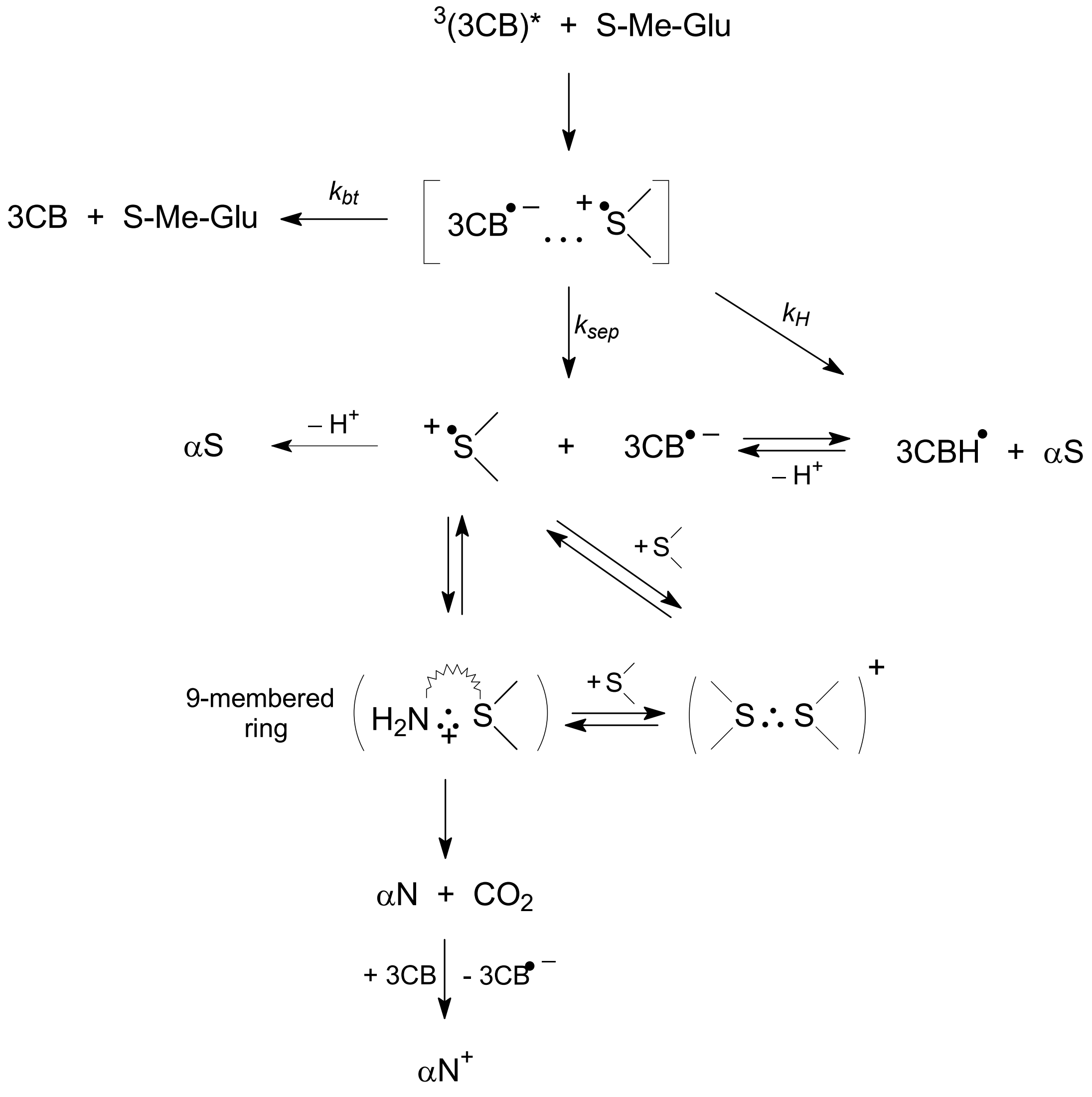

Scheme SI. Mechanism of primary reactions in 3CB sensitized photooxidation of S-Me-Glu at pH = 11.2.

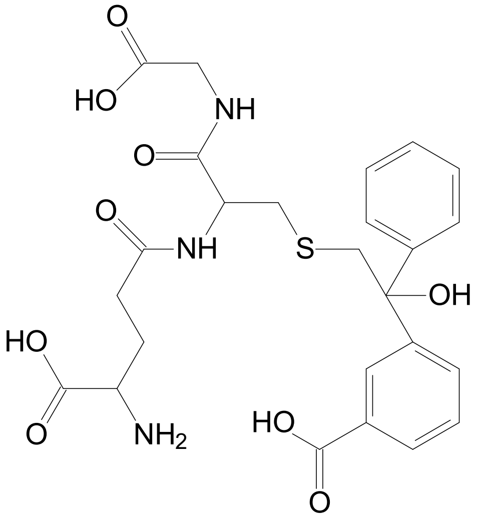

P2 αS-Sens m/z 548

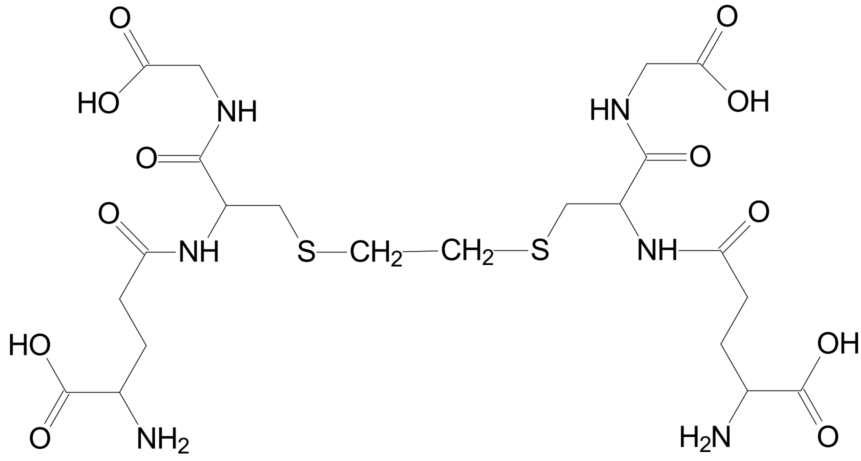

P3 αS-αS m/z 641

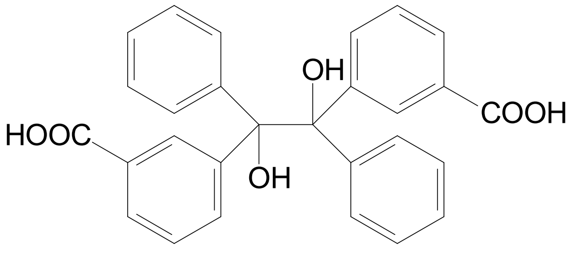

P4 Sens-Sens m/z 455

Figure S6. Suggested structures of products: **P2** m/z 548 - adduct of S-Me-Glu α-thioalkyl radical (αS) with 3CB ketyl radical; **P3** m/z 641 - αS radical dimer; **P4** m/z 455 - 3CB ketyl radical dimer.

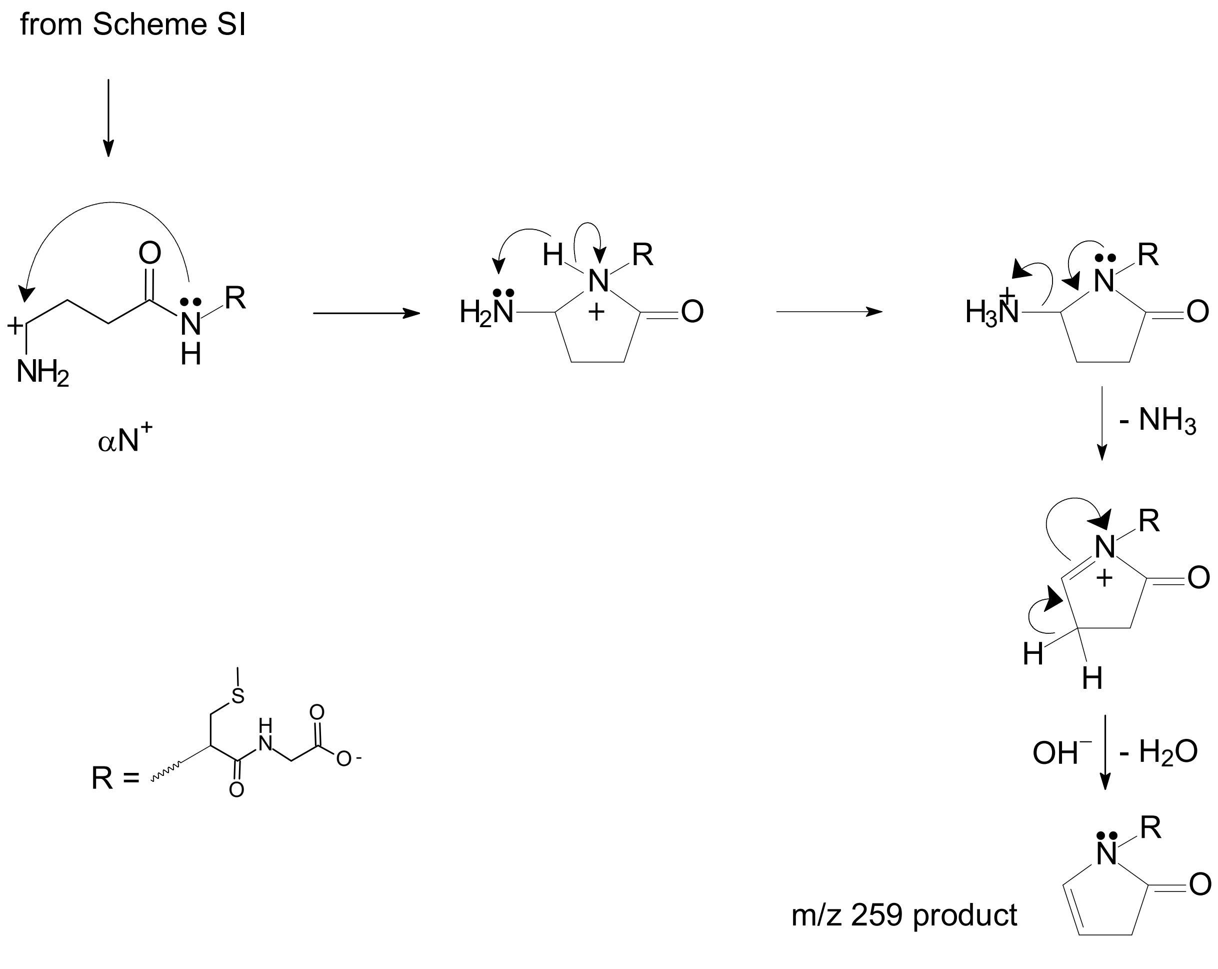

Scheme SII. Suggested mechanism of cyclic product formation from the αN⁺ derived from S-Me-Glu.

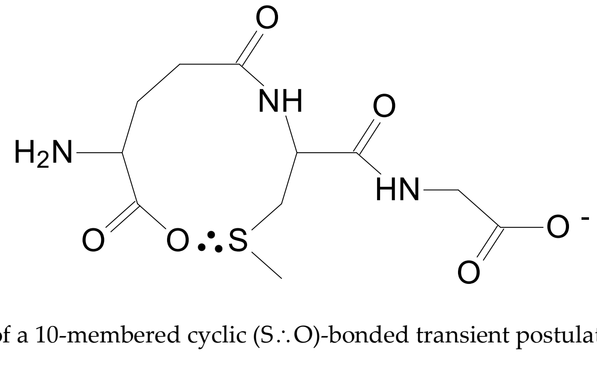

Figure S7. Structure of a 10-membered cyclic (S∴O)-bonded transient postulated in the work of [32].
